# Non-paraneoplastic autoimmune retinopathy that developed in fellow eye 10 years after onset in first eye: a case report

**DOI:** 10.1186/s12886-020-01414-z

**Published:** 2020-04-06

**Authors:** Gen Miura, Takayuki Baba, Takehito Iwase, Hisao Ohde, Atsuhiro Kanda, Wataru Saito, Susumu Ishida, Shuichi Yamamoto

**Affiliations:** 1 Department of Ophthalmology and Visual Science, Chiba University Graduate School of Medicine, Inohana 1-8-1, Chuo-ku, Chiba, 260-8670 Japan; 2 Department of Ophthalmology, Keio University, Tokyo, Japan; 3 Department of Ophthalmology, Faculty of Medicine and Graduate School of Medicine, Hokkaido University, Sapporo, Japan

**Keywords:** Autoimmune retinopathy, Non paraneoplastic autoimmune retinopathy, Alpha-enolase

## Abstract

**Background:**

Evidence-based criteria for the treatment of autoimmune retinopathy (AIR) have not been established. The pathology and clinical features of each antibody causing AIR, and its long-term course are still undetermined. We report our findings in a case of non-paraneoplastic AIR (npAIR) that developed in the fellow eye 10 years after the onset in the first eye.

**Case presentation:**

Our patient had photophobia in both eyes and a rapidly progressing visual field defect in his right eye at the initial examination. He was diagnosed with non-paraneoplastic AIR based on the clinical findings and immunoblot analyses for anti-retinal antibodies, and he was treated with steroids. Ten years later, a visual field defect developed in the fellow eye, and a diagnosis of npAIR was made. Immunoblot analyses were positive for anti-α-enolase antibodies. He was treated with steroids, immunosuppressants, and plasma exchange. However, the response to the treatment was poor and both eyes eventually became blind.

**Conclusions:**

As best we know, this is the first case report of npAIR that developed in the fellow eye over 10 years after the development in the first eye. Long-term follow-up and a search for tumor lesions are necessary in cases of npAIR. Further understanding of the long-term course of AIR can contribute to an understanding of the pathology and treatment of npAIR.

## Background

Autoimmune retinopathy (AIR) consists of a group of inflammation-mediated retinal disorders which are characterized by a reduction in vision, defects in the visual field, dysfunction of the photoreceptors, and presence of antiretinal antibodies. Cancer-associated retinopathy, first reported in 1976, is characterized by vision reduction due to photoreceptor degeneration and the presence of a cancerous lesion [1]. AIR without the detection of a malignancy is called non-paraneoplastic retinopathy (npAIR), and it was first reported in 1997 [2]. Despite the many case reports since this report, the diagnosis, management, and treatment of AIR is still a challenge because the clinical diagnostic criteria and treatment methods have not been definitively established. In addition, there are still many unanswered questions on the long-term prognosis of npAIR.

Thus, the purpose of this report is to present our findings in a case of npAIR that developed in the fellow eye 10 years after the onset of npAIR in the first eye.

## Case presentation

### Development of npAIR in first eye

A 51-year-old man presented with a history of a progressive loss of his peripheral visual field in the right eye and photophobia in both eyes that was first noted in February 2003. He had been treated with two courses of 1000 mg intravenous methylprednisolone for 3 days by his previous physician. After those treatments, he was referred to our hospital in April 2005.

**Fig. 1:**
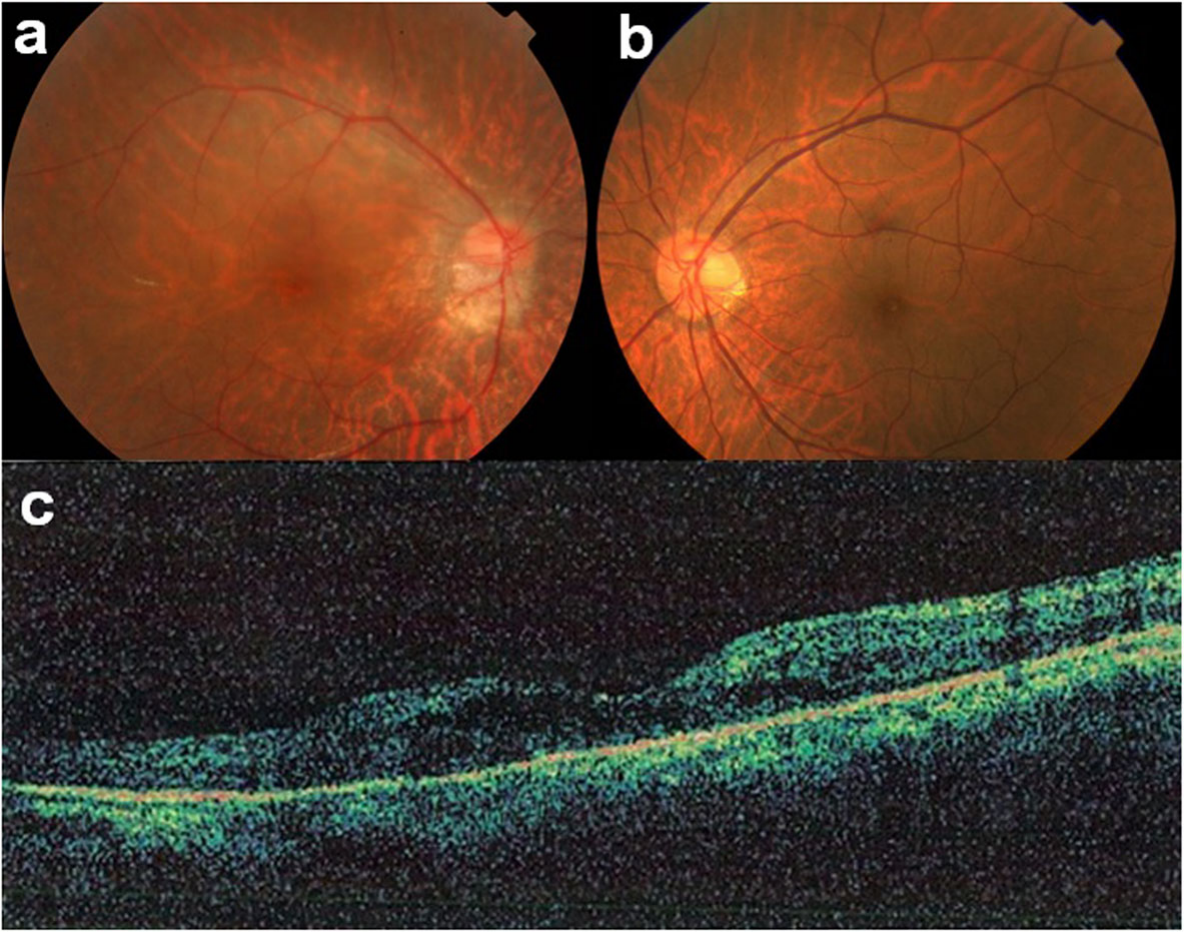
Fundus photographs of right and left eyes (**a**, **b**) and OCT images of the right eye (**c**) at initial visit in 2005. Optic disc swelling is present only in the right eye. Retinal vessels in the right eye are narrower than that of the fellow eye. Outer retinal bands are not clearly seen and edema is present in the macular area of the right eye in the OCT image

**Fig. 2:**
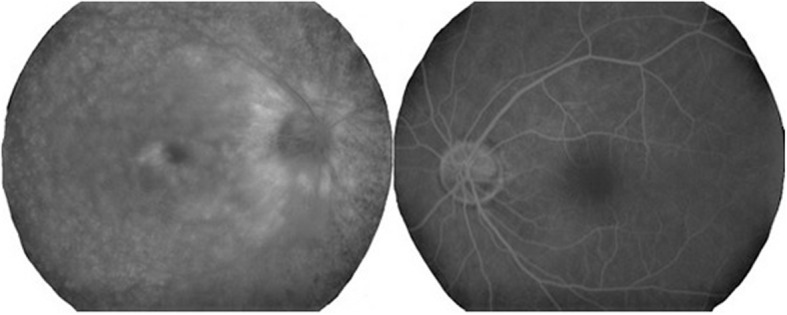
Fluorescein angiogram (FA) at the initial visit shows window defects corresponding to the retinal pigment epithelial atrophy and staining of the parafoveal tissue and leakage from the right optic disc

**Fig. 3:**
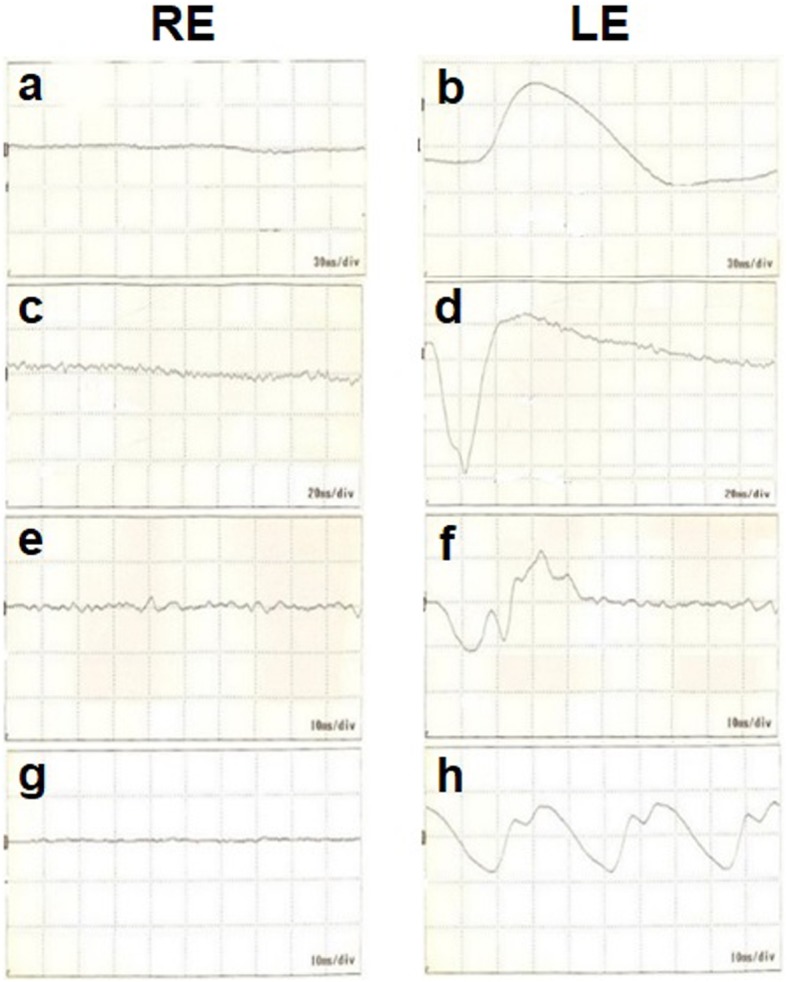
ERG waveforms at initial visit. **a** and **b**, dark-adapted 0.01; **c** and **d**, dark-adapted 3.0; **e** and **f**, light-adapted 3.0; g and h, light-adapted 30 Hz flicker. a, c, **e**, and g ERGs from right eye; **b**, **d**, **f**, and **h** ERGs from left eye

**Fig. 4:**
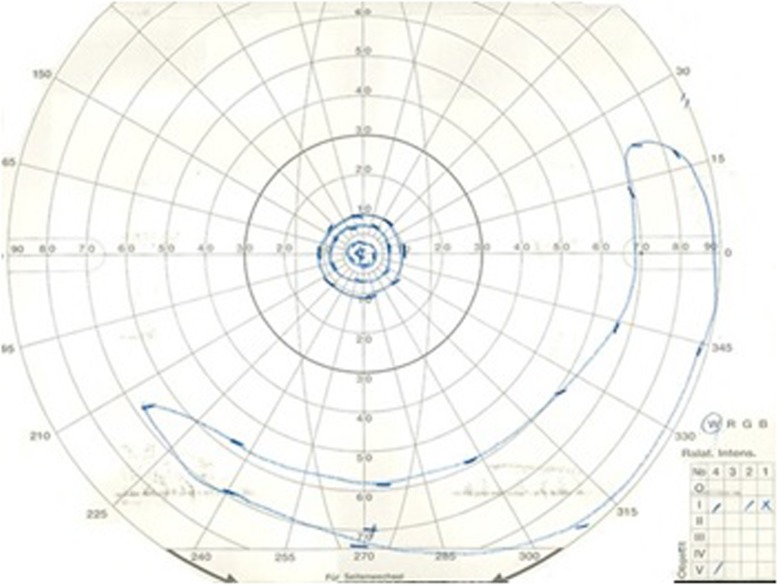
Goldmann perimetry at initial visit shows a peripheral visual field loss in the right eye

Our initial examination in 2005 showed that he had no personal or family history of ocular or autoimmune diseases. His best-correlated visual acuity (BCVA) was 20/25 in the right eye and 20/16 in the left eye. A swelling of the optic disc was detected but only in the right eye. The diameter of the retinal vessels in the fundus photographs was narrower in the right eye than that of the fellow eye (Fig. 1a-b), and the optical coherence tomographic (OCT; Fig. 1c) images showed that the outer retinal bands in the right eye were not clear and edema was present in the macula. Fluorescein angiography (FA) demonstrated window defects corresponding to the site of the retinal pigment epithelial atrophy. FA also showed staining of the parafoveal tissue and leakage from the right optic disc (Fig. 2). Electroretinograms (ERGs) were non-recordable from the right eye and normal in the left eye (Fig. 3). Goldmann perimetry detected a peripheral visual field loss in the right eye (Fig. 4). Immunoblot analyses detected no anti-retinal antibodies. During the entire course, no tumor lesions were found by systemic examinations including gastrointestinal endoscopy, computed tomography (CT), and positron emission tomography CT (PET-CT). Because the search for anti-retinal antibodies was negative, npAIR was suspected based on the clinical findings [3]. The response to steroid treatment was poor, and the vision in his right eye decreased to no light perception.

### Development of npAIR in fellow eye

**Fig. 5:**
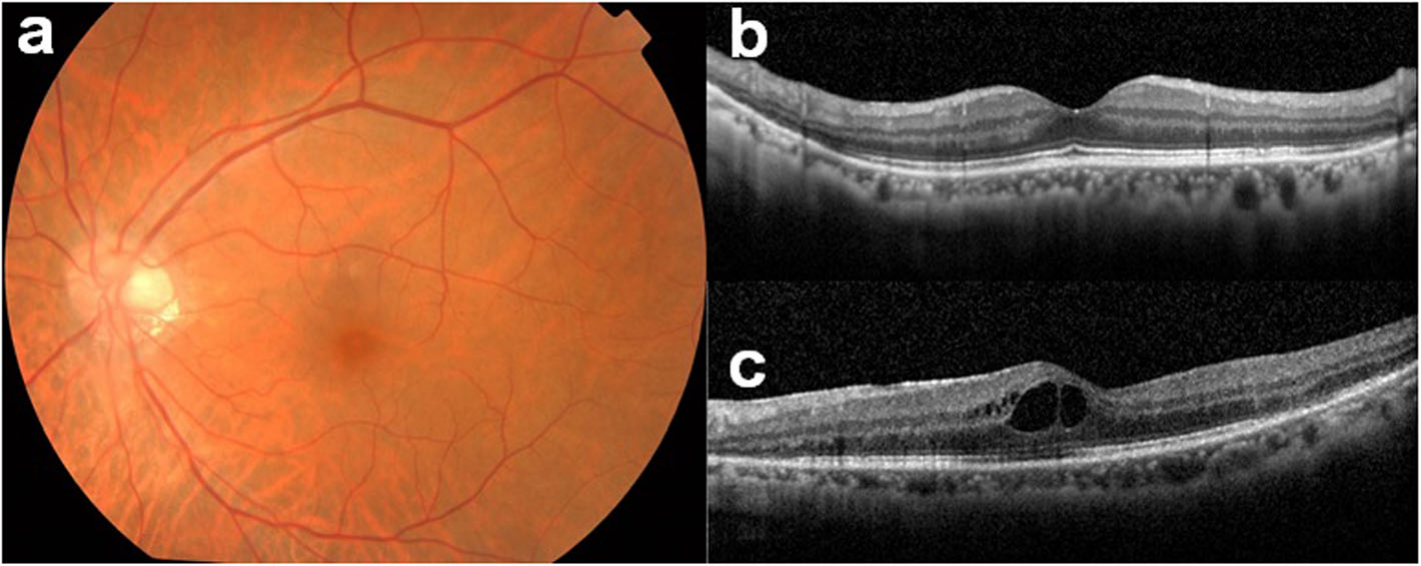
Fundus photographs of the left eye (**a**), OCT of the left eye (**b**) at onset of retinopathy of the fellow eye. OCT of the left eye (**c**) at 9 months after second onset of symptoms. Fundus photograph of the left eye showed no obvious abnormal findings. The reduction in the length of the ellipsoid zone (EZ) and partial discontinuity of the interdigitation zone can be seen in the OCT image. OCT also shows macular edema of the left eye 9 months after the second onset

**Fig. 6:**
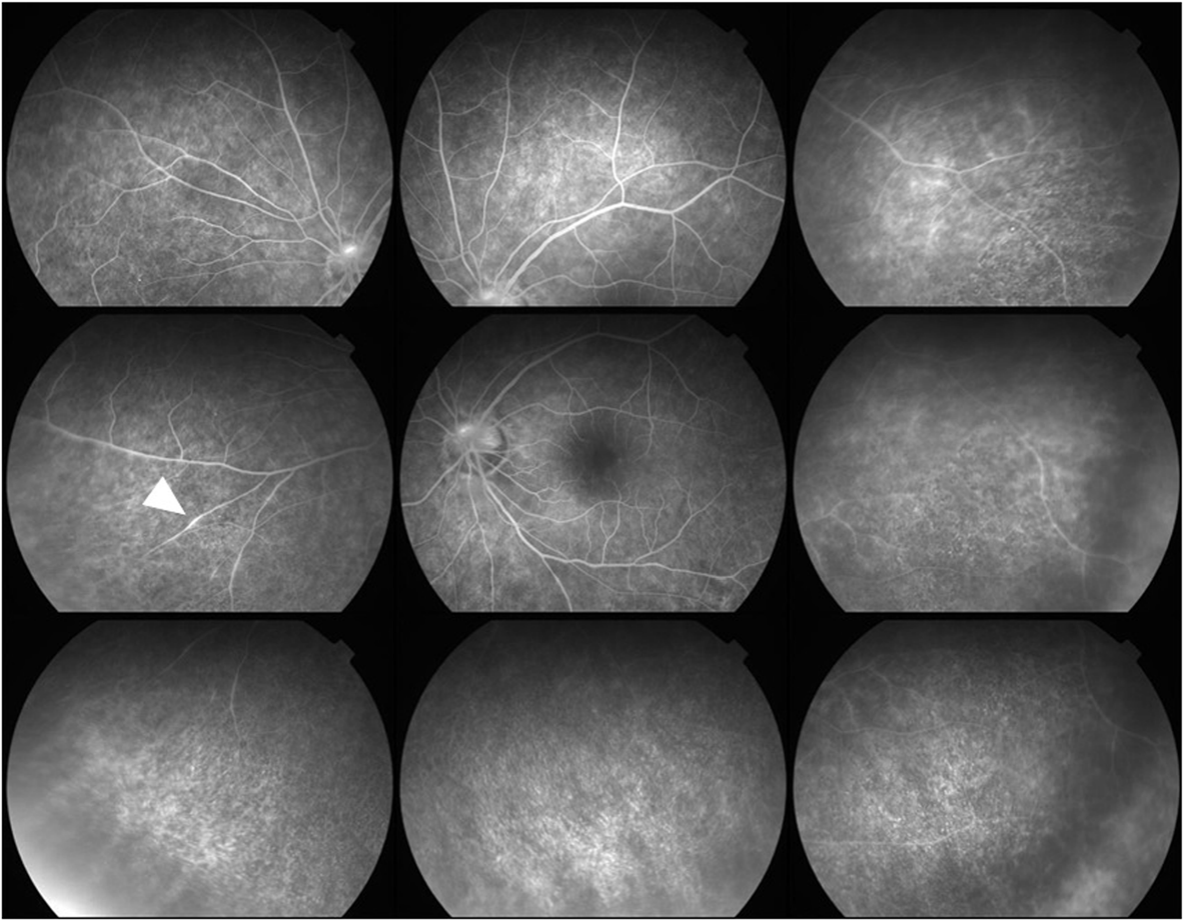
FA at second onset shows peripheral window defects and leakage from the retinal vessel (arrowhead) of the left eye

**Fig. 7:**
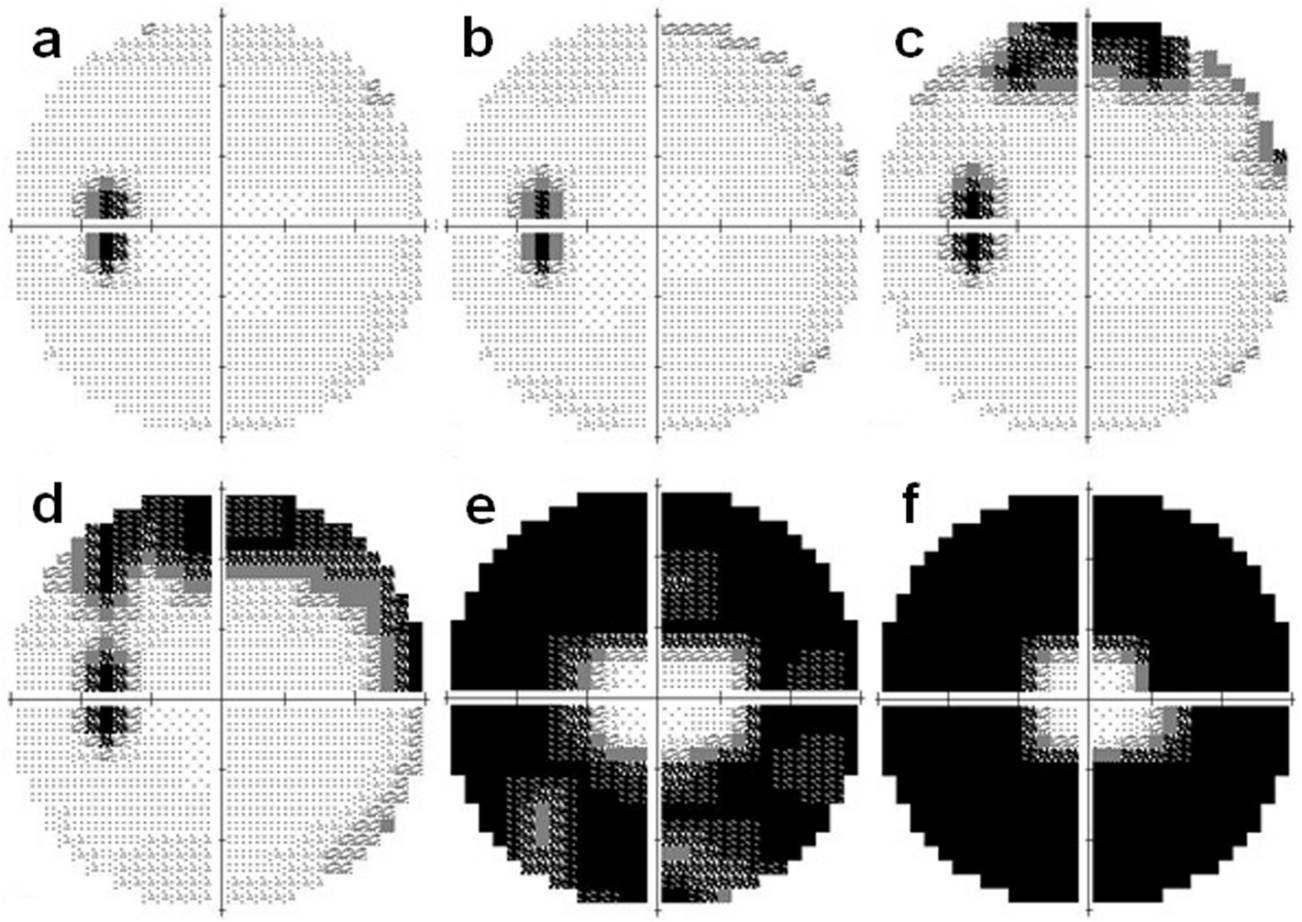
HVF 30–2 of the left eye at November 2012 (**a**), June 2013 (**b**), second onset at January 2014 (**c**), May 2014 (**d**), October 2014 (**e**) and April 2015 (**f**). The visual field defects progressed rapidly to a ring scotoma

**Fig. 8:**
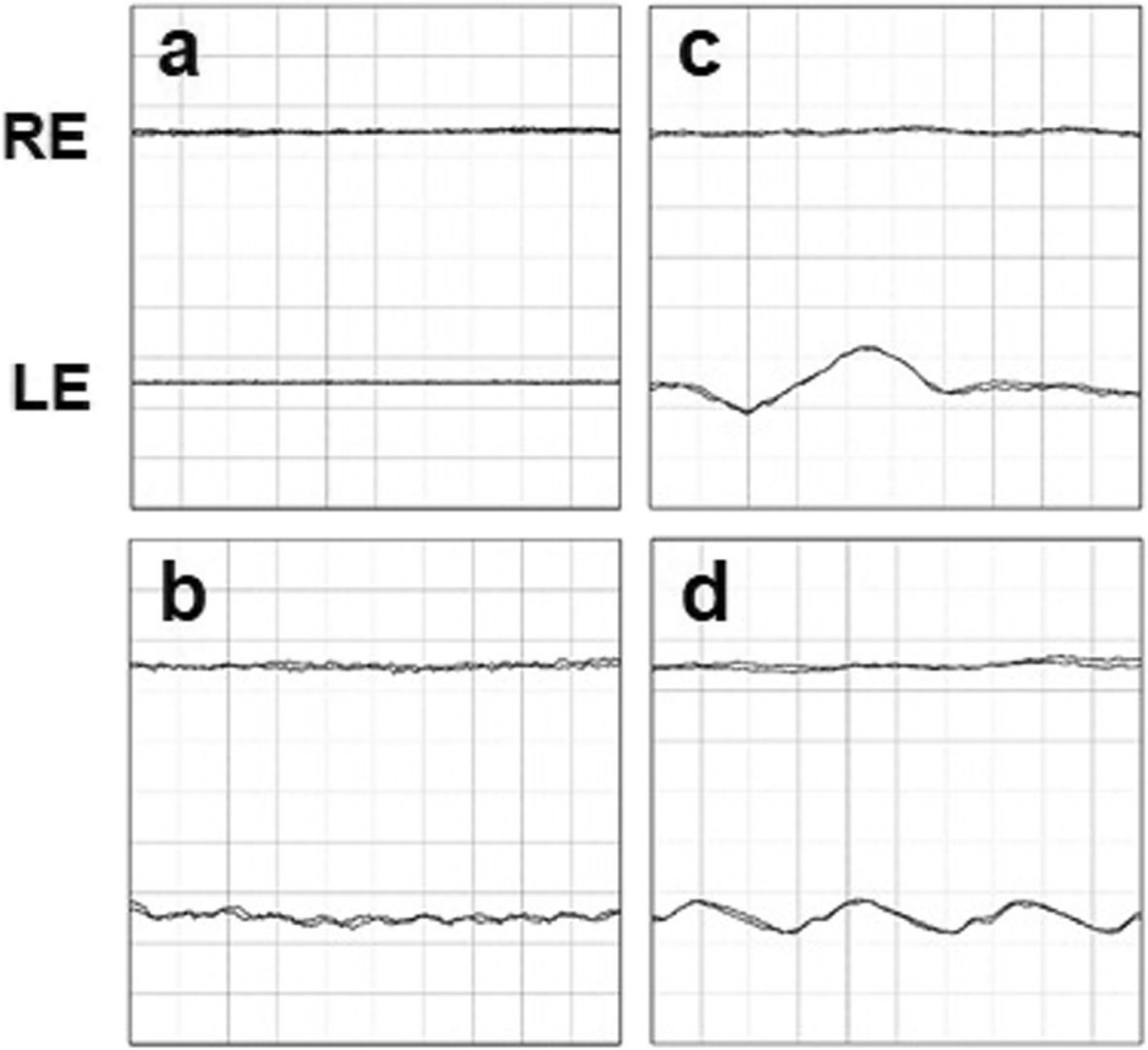
ERG waveforms at second onset. **a**, dark-adapted 0.01; **b**, dark-adapted 3.0; **c**, light-adapted 3.0 ERG; **d**, light-adapted 30 Hz flicker. The upper line shows the result of the right eye and the lower line shows the result of the left eye

Although the left eye had photophobia since the initial visit in 2005, no abnormal subjective or objective findings were observed until 2014. He noticed an upper visual field defect in the left eye in January 2014, ten years after the first onset in the right eye. The BCVA was 20/16 in the left eye at this time. Fundus photographs showed no obvious abnormal findings in the left eye (Fig. 5a). However, a reduction in the length of the ellipsoid zone (EZ) and a partial discontinuity of the interdigitation zone (IZ) were seen in the OCT images (Fig. 5b). In addition, OCT showed macular edema in the left eye in October 2014 (Fig. 5c). FA detected peripheral window defects in the left eye (Fig. 6). No visual field abnormality was observed until 2013, however Humphrey field analyzer (HFA) 30–2 examination in January 2014 showed an upper visual field defect. The visual field defect progressed rapidly to a ring scotoma (Fig. 7). Dark-adapted ERGs of the left eye were non-recordable. The amplitude of the light-adapted and flicker ERGs of the left eye were reduced with prolonged implicit times (Fig. 8). Immunoblot analyses were positive for anti-α-enolase antibodies. A complete examination including gastrointestinal endoscopy, CT, and PET-CT was performed again, and no tumor lesions were detected. No inflammation was observed in the anterior segment or the vitreous at any time.

Two courses of 1000 mg intravenous methylprednisolone for 3 days was given based on a diagnosis of npAIR. After the steroid pulse therapy, oral administration of prednisolone of 30 mg/day and oral immunosuppressant were initiated and were continued. The symptoms gradually worsened in spite of these treatments. Therefore, six plasmapheresis treatments were administered from June 2016. However, the response to these treatments was poor, and the vision in his left eye eventually became no light perception. At that time, funduscopic examination of the fellow eye demonstrated attenuation of the retinal vessels, and fundus autofluorescence of the fellow eye demonstrated retinal pigment epithelial atrophy in the midperipheral area.

## Discussion and conclusions

It has been reported that photophobia and photosensitivity are characteristic symptoms of AIR [4]. Our case had bilateral photophobia since the onset in the first eye. However, AIR usually develops bilaterally [5] which was different from our case.

Enolase is a 46-kDa glycolytic enzyme that is expressed in both the retina and the optic nerve [6]. Anti-enolase antibodies have been reported to be positive not only in systemic autoimmune diseases [7] but also in patients without a tumor and 10% of healthy subjects [8]. However, considering the reported diagnostic criteria for AIR and the clinical characteristics of α-enolase antibody-positive autoimmune retinopathy, we believe that this antibody affected the development and clinical symptoms of retinopathy in our case. The clinical manifestations of anti-α-enolase antibody-positive paraneoplastic AIR patients were reported to be relatively mild, and the progression was comparatively slow [9]. The findings in our case are consistent with these earlier cases in which α-enolase antibodies were positive and the progression was slow. The clinical features of Japanese patients with anti-α-enolase antibody-positive AIR were recently published [10]. The authors reported that OCT showed drusen of various sizes with domed-shaped hyperreflective spots under the retinal pigment epithelium corresponding to the drusen in 48% of the cases. However, neither the fundus photographs nor the OCT images showed any obvious drusen in our case. The authors also reported that the BCVA improved or was maintained in 80% of the eyes during the follow-up period. However, their study period was at least two months, making it difficult to compare their findings with that of our case. Saito et al. presented a case with small cell lung carcinoma that developed bilateral neuroretinitis with unilateral focal outer retinitis that was positive for autoantibodies against recoverin, CRMP-5, and α-enolase [11]. They reported that the recoverin-mediated autoimmune retinopathies and ophthalmic findings in their case had inflammatory features. Although similar to our case by the presence of inflammation, the response to treatment was different. It is not known why there was a difference in the response to treatment, however their case was associated with a tumor which could be treated. Adamus et al. reported that a rebound of the anti-recoverin autoantibody titer was associated with exacerbations of the visual symptoms. However, anti-recoverin antibody was not detected throughout the clinical course of our case [12]. In addition, Ferreyra et al. reported that there was an improvement in vision in only 19% of npAIR without CME and in 25% of npAIR with CME after treatment [3]. The authors also showed that the subgroup most responsive to immunosuppression treatment was the paraneoplastic AIR group and the least responsive was the npAIR group [13]. A case of unilateral TRPM1-positive CAR associated with adenocarcinoma of the right ovary has been reported [14]. The patient was treated with rituximab, monoclonal antibody, and corticosteroids which resulted in a visual acuity of 20/20, symptomatic improvements, and normalization of the ERGs. Although there are differences from our case, such as the type of antibody, the presence of a tumor or method of treatment, further long-term follow-up of the patient is necessary. A case of CAR more similar to our case was reported by Saito et al. [15]. Goldmann perimetry and ERGs of their case showed retinitis pigmentosa–like findings in the right eye and a normal appearance in the left eye at the first onset. Eight years later, the left eye also presented with a visual field defect and ERG abnormalities, and immunoblot analyses detected anti-recoverin antibodies. The differences from our case are that in their case a bronchioloalveolar carcinoma was detected and treated, that anti-recoverin antibodies were detected, and that the visual prognosis was good only with 40 mg/g of oral administration of prednisolone. Their case report indicates that regular screening for tumors is necessary even in cases where no tumor was detected as in our case.

It is not clear why the disease initially developed in only one eye, and why it took 10 years to develop in the fellow eye. In addition, we could not determine why the response to treatment was poor. Our case was not treated with biologics such as anti-CD20 monoclonal antibody, e.g., Rituximab, or intravenous immunoglobulin. The treatment consisted of only steroids at the initial onset. Therefore, the clinical course might have been different if further treatments had been given at that time.

In summary, we presented our findings in a case of npAIR that developed in the fellow eye more than10 years after its onset in the first eye. Our case had differences in the clinical findings, characteristics, and course compared to earlier cases. Because our case was resistant to treatment and had poor visual functional outcome, cases such as ours require careful follow-up. There is still no established evidence-based treatment for cases of npAIR, and it varies among physicians, regions, and facilities. Sharing the history of this case will help to reaffirm the importance of follow-up examinations in patients with npAIR and in considering treatment options.

## Data Availability

All the data supporting our findings are contained within the manuscript.

## Abbreviations

AIR: Autoimmune retinopathy
npAIR: Non-paraneoplastic autoimmune retinopathy
BCVA: Best-corrected visual acuity
OCT: Optical coherence tomography
FA: Fluorescein angiography
ERG: Electroretinogram
CT: Computed tomography
PET-CT: Positron emission tomography
EZ: Ellipsoid zone
IZ: Interdigitation zone
HFA: Humphrey field analyzer
CME: Cystoid macular edema
